# P-660. Exploring the Association Between *Helicobacter Pylori* Infection and Bronchiectasis: A Meta-analysis

**DOI:** 10.1093/ofid/ofae631.857

**Published:** 2025-01-29

**Authors:** Rukesh Yadav

**Affiliations:** Maharajgunj Medical Campus, Institute of Medicine, Tribhuvan University, Kathmandu, Bagmati, Nepal

## Abstract

**Background:**

*Helicobacter Pylori* (*H. pylori*) is known to cause various malignancies, and autoimmune disorders. However, the potential association between *H. pylori* and bronchiectasis has been not analyzed in the literature before. Thus, we aimed to explore the potential association between bronchiectasis and *H. pylori*.

**Figure:**
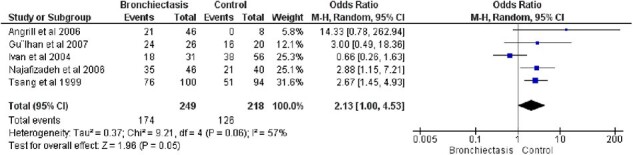

Forest plot showing the odds of association between H pylori seropositivity and bronchiectasis

**Methods:**

Pubmed, Google Scholar and Embase databases were searched from inception till January 2024 for studies that investigated *H. pylori* seropositivity, anti H *pylori* IgG level, urea breath test (UBT) positivity, polymerase chain reaction (PCR) and culture to detect *H pylori* in gastric juice (GJ) and bronchoalveolar lavage fluid (BALF) in bronchiectasis and matched healthy controls. Outcomes included odds ratio of association between *H. pylori* and bronchiectasis, detection rate of *H. pyroli* in BALF, GJ or bronchial biopsies, standardarized mean difference (SMD) of serum anti H *pylori* IgG level between the bronchiectasis patients and the healthy controls. The analysis was performed in Revman 5.4 software.

**Figure:**
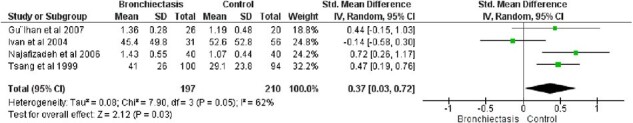

Forest plot showing serum anti H. pylori IgG levels between bronchiectasis and healthy controls

**Results:**

Five studies comprising 455 participitants were included in this analysis. *H. pylori* seropositivity was nearly significantly associated with increased odds of having bronchiectasis. [Odd’s ratio=2.13, Confidence Interval (CI)=1.00-4.53, I^2^=53%, P value =0.05]. Moreover, the serum anti *H. pylori* IgG levels were significantly higher in bronchiectasis patients as compared to healthy controls . [SMD=0.37, CI=0.03-0.72, I^2^=62%, P value =0.03]. However, none of the studies isolated *H. pylori* from bronchial biopsies in both the bronchiectasis patients and the healthy controls. Interestingly, a study by Teke et al. found that nine patients (22%) of the 41 bronchiectasis patients and three patients (18.8%) of the 16 the controls had positive BALF on PCR, but this was not significantly different between the two groups. The other study by Gulhan et al. however did not find *H. pylori* on BALF PCR in any of the 26 bronchiectasis patients.

**Conclusion:**

Though the *H pylori* seropositivity was associated with bronchiectasis, further future studies of larger sample sizes need to be conducted. Further, newer methods to detect H. pylori in bronchiectasis patients need to be explored to prove the association owing to higher H. *pylori* seropositivity in the general population.

**Disclosures:**

**All Authors**: No reported disclosures

